# Systems biology analysis reveals new insights into invasive lung cancer

**DOI:** 10.1186/s12918-018-0637-z

**Published:** 2018-12-14

**Authors:** Dan Li, William Yang, Carolyn Arthur, Jun S. Liu, Carolina Cruz-Niera, Mary Qu Yang

**Affiliations:** 1 MidSouth Bioinformatics Center and Joint Bioinformatics Ph.D. Program, University of Arkansas at Little Rock and University of Arkansas for Medical Sciences, 2801 S. University Avenue, Little Rock, AR 72204 USA; 2 Department of Computer Science, Carnegie Mellon University School of Computer Science, Pittsburgh, PA 15213 USA; 3 Department of Genetics, Yale University, 333 Cedar Street, New Haven, CT 06520 USA; 4 Department of Statistics, Harvard University, One Oxford Street, Cambridge, MA 02138 USA; 5 Department of Information Science and Department of Computer Science, Member of United States National Academy of Engineering, George Washington Donaghey College of Engineering & IT, University of Arkansas at Little Rock, 2801 S. University Avenue, Little Rock, AR 72204 USA

## Abstract

**Background:**

Adenocarcinoma in situ (AIS) is a pre-invasive lesion in the lung and a subtype of lung adenocarcinoma. The patients with AIS can be cured by resecting the lesion completely. In contrast, the patients with invasive lung adenocarcinoma have very poor 5-year survival rate. AIS can develop into invasive lung adenocarcinoma. The investigation and comparison of AIS and invasive lung adenocarcinoma at the genomic level can deepen our understanding of the mechanisms underlying lung cancer development.

**Results:**

In this study, we identified 61 lung adenocarcinoma (LUAD) invasive-specific differentially expressed genes, including nine long non-coding RNAs (lncRNAs) based on RNA sequencing techniques (RNA-seq) data from normal, AIS, and invasive tissue samples. These genes displayed concordant differential expression (DE) patterns in the independent stage III LUAD tissues obtained from The Cancer Genome Atlas (TCGA) RNA-seq dataset. For individual invasive-specific genes, we constructed subnetworks using the Genetic Algorithm (GA) based on protein-protein interactions, protein-DNA interactions and lncRNA regulations. A total of 19 core subnetworks that consisted of invasive-specific genes and at least one putative lung cancer driver gene were identified by our study. Functional analysis of the core subnetworks revealed their enrichment in known pathways and biological progresses responsible for tumor growth and invasion, including the VEGF signaling pathway and the negative regulation of cell growth.

**Conclusions:**

Our comparison analysis of invasive cases, normal and AIS uncovered critical genes that involved in the LUAD invasion progression. Furthermore, the GA-based network method revealed gene clusters that may function in the pathways contributing to tumor invasion. The interactions between differentially expressed genes and putative driver genes identified through the network analysis can offer new targets for preventing the cancer invasion and potentially increase the survival rate for cancer patients.

**Electronic supplementary material:**

The online version of this article (10.1186/s12918-018-0637-z) contains supplementary material, which is available to authorized users.

## Background

Lung Adenocarcinoma in situ, is a pre-invasive non-small-cell lung cancer (NSCLC) lesion. The early diagnosed and appropriately treated AIS patients often have quite high survival rate (almost 100%) [1]. A fraction of AIS can develop into invasive cancer. The 5-year survival rate for the invasive lung cancer is decreased to 4% on average [2]. Presently, about 70% of the lung cancer cases are diagnosed at the invasive stage [3]. Several studies have investigated the progression of the lung cancer invasion [4, 5]. For example, Min et al. followed a case of lung cancer that evolved from a pure ground-glass opacity nodule into an invasive adenocarcinoma for 10 years and studied the growth and aggressiveness of the lung cancer [6]. Another study indicated that the protein transforming growth factor-ß (TGF-ß) induces epithelial-mesenchymal transition (EMT) in lung cancer cells and further mediates the tumor migration and invasion [7]. A recent study investigated RNA sequencing (RNA-seq) data generated from AIS and invasive lung cancer tissue samples and identified several genes that potentially involved in the progression from AIS to invasion [1]. However, the regulations of the genes and the underlying molecular mechanisms that govern the invasion progression are not well characterized.

We developed a Genetic Algorithm based method to infer lung cancer invasion-related gene networks. We first identified a set of genes that were differentially expressed in invasive lung adenocarcinoma by comparing gene expression alterations in normal, AIS, and invasive tumor tissues based on a RNA-seq dataset [1]. We found that these genes showed consistent expression patterns in a LUAD dataset from The Cancer Genome Atlas. Then, we employed a global optimal search algorithm to construct subnetworks for each invasive differentially expressed gene by integrating gene expression, protein-protein interactions (PPIs), protein-DNA interactions and lncRNA regulations. Further incorporation of driver mutation information, we revealed 19 core subnetworks that contained invasive specific genes and putative driver genes. These subnetworks can lead us to the discovery of new pathways responsible for invasive tumor progression.

**Fig. 1:**
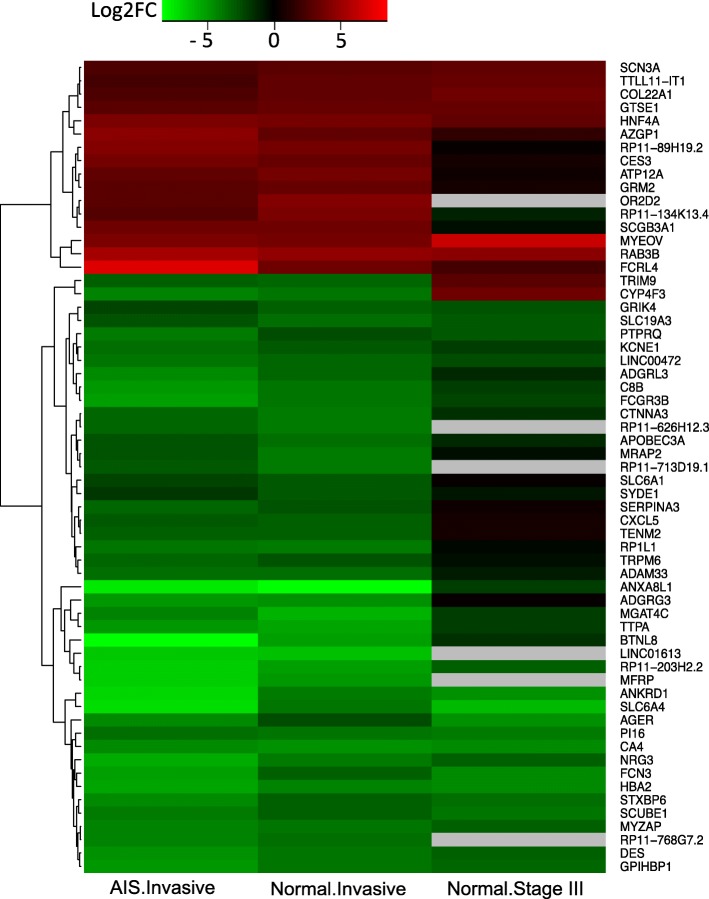
Expression alterations of invasive-specific genes. The expression patterns (log2 fold change) of the invasive-specific genes identified by the comparison between normal and invasive, AIS and invasive samples, and TCGA LUAD normal and stage III samples

## Results

### Identification of invasive specifc genes

The RNA sequencing data of normal, AIS, and invasive tissue sampes for six lung cancer patients were collected from Gene Expression Omnibus (GSE52248) [1]. Differential expression analysis revealed diverse gene expression change patterns. We found that 98 genes were significantly differentially expressed between AIS and invasive (|FC| > 2 and FDR < 0.05). Among these genes, 61 were also differentially expressed in normal and invasive comparison (|FC| > 2 and FDR < 0.05). Hence, we considered these 61 genes to be lung invasive-specific differentially expressed genes (DEGs), which consisted of 52 protein-coding genes and 9 lncRNAs (Additional file 1: Table S1). The expressions of the invasive-specific genes are able to separate the 18 tissue types with different phenotypes by hierarchical clustering (Additional file 2: Figure S1), only one invasive tissue sample was misclustered. This sample was clustered together with an AIS sample from the same patient. This misclustered case might be related with the sample collection. We further validated these invasive-specific genes on an independent RNA-seq data for 59 normal and 84 stage III lung adenocarcinoma (LUAD) tissue samples obtained from the TCGA project. The hierarchical clustering based on the expression levels of invasive specific genes demonstrated two unique tissue clusters, normal and stage III LUAD, and only 5 of 143 (3.5%) tissue samples were mis-clustered (Additional file 3: Figure S2).

Of the 61 invasive-specific genes, 16 were upregulated and 45 were downregulated in the invasive tissues compared to their expression levels in normal and AIS tissues (Fig. 1). Similar regulation patterns were observed in normal vs. stage III LUAD (TCGA) comparison with a few exceptions. In the TCGA samples, *TRIM9* and *CYP4F3* were expressed in the opposite manner, and the other two protein-coding genes and four lncRNAs were not differentially expressed (Fig. 1). The functional annotation by DAVID [8] of the upregulated and downregulated gene sets revealed several cancer related biological processes. The inflammatory response (P-value = 0.054, downregulated genes) and negative regulation of cell growth (P-value = 0.063 upregulated genes) were enriched, indicating their roles in invasive cancer development (Additional file 4: Table S2).

### Putative driver somatic mutations

The somatic mutations were identified using MuTect2 (Table 1, Methods) based on paired RNA-seq data (normal and AIS, normal and invasive). We found a total of 271,064 and 273,292 significant somatic mutations in AIS and invasive lung tissues respectively. Then we employed Cancer-specific High-throughput Annotation of Somatic Mutations (CHASM) to predict driver mutations [9]. Our results showed that 362 of the 6445 mutated genes in AIS tissues reported as driver genes, while 411 of the 6509 mutated genes in invasive tissues were identified as drivers (CHAMS score > 0.8 and P-value < 0.05, Methods).

**Table 1:** Identification of somatic mutations and putative driver genes in LUAD

	AIS samples			Invasive samples		
Data sets	Somatic mutations (PASS)	Mutated genes	Putative driver genes	Somatic mutations (PASS)	Mutated genes	Putative driver genes
AIS lung cancer samples	271,064	6445	362	273,292	6509	411
TCGA stage III				58,985 (85 samples)	10,743	201

Meanwhile, the somatic mutation data of stage III TCGA lung adenocarcinoma were collected. Stage III tumors usually have increased size, extent, or degree of penetration, but no distant metastasis [10]. We found 201 significant putative LUAD driver genes based on TCGA stage III cases (CHASM score > = 0.8 & P-value < 0.05). There was a total of twenty-seven common putative driver genes between the TCGA stage III dataset and AIS lung cancer dataset. The two patient datasets were independent. The relative low concordat rate (13.4% and 6.6% of TCGA stage III and AIS datasets respectively) here may attribute to the heterogeneity of the disease. Out of these putative driver genes, 68.2% (137/201) were consistently highly expressed (median FPKM > 1) in both the normal and invasive samples, while only 29.9% (60/201) were differentially expressed genes.

### Lung adenocarcinoma invasion associated gene subnetworks

Next, we constructed subnetworks to explore the relationships between putative driver genes and differentially expressed genes in the disease. Our analysis showed that the majority invasive-specific genes had no driver mutations (98.4%, 60/61), *ADGRL3* (CHASM score = 0.824, P-value = 0.0044) was the only predicted LUAD driver gene, while most (70.1%) putative driver genes were not differentially expressed. Hence, the subnetworks built based on the integration of mutations and expression profiles can connect genotype with transcription and potentially reveal novel pathways in the disease. We then adopted GA to search for the gene groups that were associated with lung cancer invasion. Based on the fitness score, GA searches the optimal resolutions in generations, potentially yielding global optimum subnetworks that discriminate tissues with different phenotypes.

**Fig. 2:**
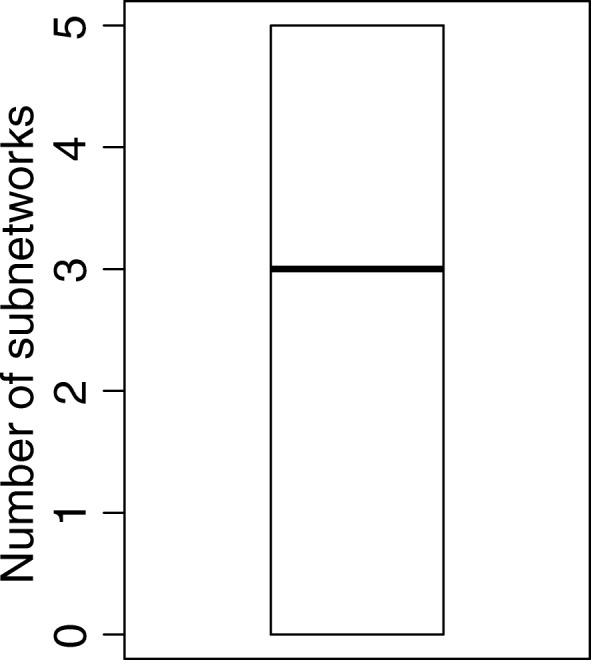
The distribution of the number of the subnetworks associated with each of the LUAD invasion seed genes

We used each invasive-specific differentially expressed gene as a seed for the subnetwork construction. All the genes directly or indirectly interacted with the seed genes through PPIs and protein-DNA interactions formed the original chromosome which is a potential solution for GA (Methods). The indicators, 1 and 0 values, indicate whether the relative gene would be collected or not. For each generation of GA, the top 5% of the chromosomes with the smallest fitness scores were kept and passed no the next generation. The fitness score here was calculated based on the mutual information. The LUAD seed genes were always kept in the gene set for calculating the fitness scores. Compared to the greedy algorithm, the GA search resulted in the subnetworks that can better distinguish invasive LUAD from normal cases. We used 500 generations in GA searching. Our data showed that no further improvement was observed by increasing the number (around 300) of generations (Additional file 5: Figure S3). Among the final networks identified by GA, some seed genes were associated with multiple subnetworks with the same smallest fitness score (Fig. 2). On the other hand, three seed genes had no interacted genes, either by PPI or protein-DNA interaction. Presently, neither PPI nor protein-DNA interaction information were available for the lncRNAs. Hence, the interactions between lncRNAs and genes were based on the results from the GENIE3 [11] for the construction of the subnetworks using lncRNAs as seed (Methods). Furthermore, we generated a single core subnetwork that was associated with each of the seed genes (Methods). Nineteen (31.1% of 61 seed gene) core subnetworks that contained at least one predicted driver gene were revealed (Additional file 6: Table S3). Two statistical tests based on random gene members and random tissue types (Methods) suggested that majority core networks were statistically significant (Additional file 7: Figure S4).

**Fig. 3:**
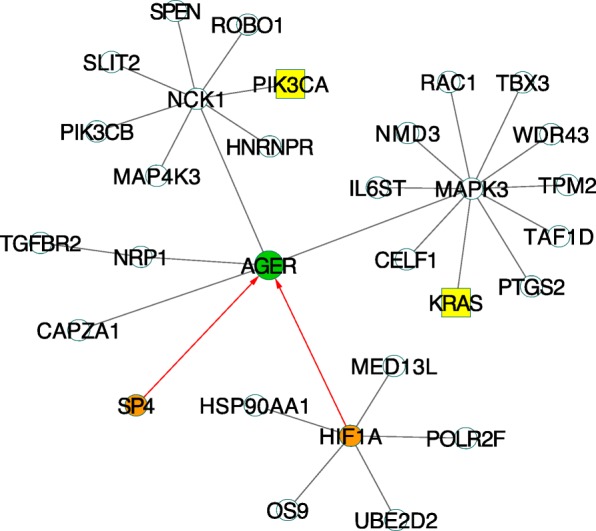
The core subnetwork of the seed gene *AGER*. *AGER* was under-expressed in invasive tumor cases. *KRAS* and *PIK3CA* were driver genes (yellow and square) predicted by CHASM. *SP4* and *HIF1A* were lung cancer specific transcription factors (orange) which regulated the expression of *AGER*

*AGER*, an invasive-specific gene, is associated with inflammatory response. Inflammation is an important factor of cancer development including lung cancer. The core subnetwork of *AGER* that consisted of 30 genes with two predicted driver genes and two transcription factors was constructed (Fig. 3). The functional analysis of this subnetwork using DAVID identified several cancer-related KEGG pathways. The top enriched pathway was the VEGF signaling pathway (P-value = 1.4E-6 and adjust P-value = 1.8E-4). The VEGF pathway has been reported to regulate tumor angiogenesis and drive the renal cell carcinoma progression [12, 13]. *KRAS* was a putative lung cancer driver gene. This gene interacted with *MAPK3* and indirectly interacted with *PTGS2, RAC1*, and *AGER* in the core subnetwork (Fig. 3). In the VEGF signaling pathway [14], *KRAS* locates at the upstream of *MAPK3* and they are both involved in the function of cell proliferation (Additional file 8: Figure S5). Thus, these invasive-specific based subnetworks (Additional file 9: Figure S6) can lead to identifying novel pathways involving in cancer invasive process.

**Fig. 4:**
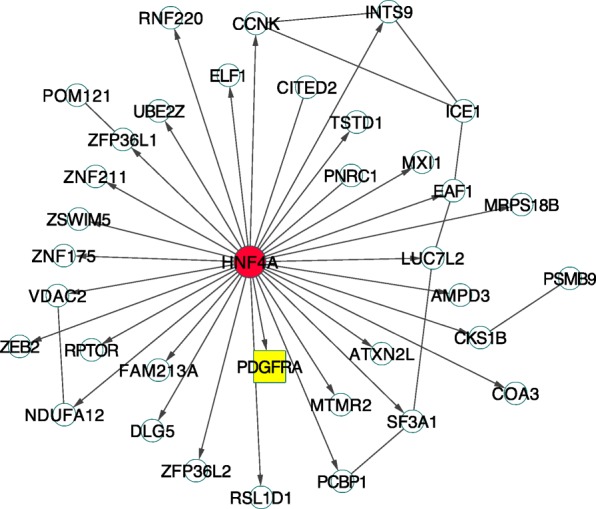
The core subnetwork of the seed gene *HNF4A*. The gene *HNF4A* is a lung adenocarcinoma related transcription factor (TF). *PDGFRA* was predicted as a lung cancer driver gene

*HNF4A* was another invasive-specific gene suggested by our study. This gene is one of the best-known tumor suppressors in liver and pancreas [15] and is related to the negative regulation of cell growth, a biologic process contributing to the tumor development and growth [16]. The core subnetwork associated with *HNF4A* (Fig. 4) showed its interaction with *PDGFRA,* a gene encode a cell surface tyrosine kinase receptor for members in the platelet-derived growth factor family [17]. *HNF4A* plays a role in organ development, wound healing, and tumor progression. Overexpression of this gene potentially promotes tumor progression and indicates poor prognosis [18]. *PDGFRA* harbored somatic mutations and was predicted as a driver gene in lung invasive cancer. Thus, abnormal expression of *HNF4A* in the disease could be the consequence of the mutations in *PDGFRA*.

## Discussion

The investigation of the process of the lung cancer developing from an unfatal subtype, such as AIS, to the invasive stage provided the insights for understanding the mechanisms responsible for deterioration of the disease. We combined the two independent datasets to infer invasive specific subnetworks. The gene expression alteration patterns tend to be more robust than somatic mutations in different patient groups. Almost 98% DEGs were the same in GSE52248 and TCGA LUAD patients. However, the putative somatic driver genes only have about the 13.4% overlap rate, reflecting the high genetic heterogeneity for the disease. Two genes, *TRIM9* and *CYP4F3*, have opposite expression patterns between the two datasets which may be explained by the diverse isoform expression patterns such as *HNF4A*. Karthikeyani Chellappa, et al. found that the diverse isoforms of *HNF4A*, especially *P2-HNF4α*, showed different expression patterns in various tissue samples [19]. As a tumor suppressor, *HNF4A* is usually downregulated in tumor samples. Interestingly, this gene was over-expressed in lung invasive tumor samples than normal of both GSE52248 and TCGA data.

The size of the chromosome of GA affects the optimal solution that the algorithm is able to find. Here, the size of the chromosome equals to the number of the candidate genes which directly or indirectly interact with the seed genes. The maximum searching distance from the seed gene was three for our subnetworks construction. At the outermost layer of the subnetworks, the total number of candidate genes often reached 18,000, which covered the majority human protein-coding genes (~ 23,000). Compared to the greedy algorithm, GA can identify global optimum subnetworks associated with the disease. The fitness function is an important factor for GA searching. Here, we used mutual information to calculate fitness score, which was estimated using discrete expression bins derived from continuous expression values. When the sample size is small, the number of final subnetworks can rapidly increase with less stability. Thus, for a small sample size, GA-based network construction may need a different fitness function guiding the searching process. In general, we found that a larger sample size could lead to more stable optimal gene groups.

## Conclusions

We developed a novel GA-based network construction method for inferring gene subnetworks associated with invasive lung adenocarcinoma. The method integrated gene expression, PPI, transcription factor and gene interaction, and lncRNA regulation to uncover global optimal subnetworks underlying invasive progression. The two independent patient datasets were used to derive invasive-specific differentially expressed genes. The 19 core subnetworks associated with invasive-specific genes contained at least one putative driver genes and were significantly enriched in several biological processes and pathways involved in tumor growth. These results could enhance our understanding of cancer progression, which helps to develop stategies for preventing the cancer invasion and improving the survival of cancer patients.

## Methods

### Identification of differentially expressed genes (DEGs)

RNA-seq data (GSE52248) generated from normal, AIS, and invasive tissues of six patients were downloaded from GEO. The sequencing quality was assessed by FastQC. The low-quality reads were trimmed by Trimmomatic (v0.36, LEADING:28 TRAILING:28 SLIDINGWINDOW:4:24 MINLEN:70) [20]. Tophat2 (v2.1.0) was applied for reads alignment and human genome hg38 was used as a reference genome for the alignment [21]. HTSeq-count (v0.8) [22] and Cufflinks (v2.2.1) [23] were performed for calculating the raw read-count and Fragments Per Kilobase of transcript per Million (FPKM), respectively, based on the gene annotation of Ensembl version GRCh38.87. After filitering out the unexpressed genes with median raw count equal to zero, edgeR [24] was used for differential expression analysis. The genes have the absolute fold-change greater than 2 with FDR < 0.05 were considered as significantly differentially expressed between different tissue types.

### Putative driver mutation identification

The paired RNA-seq reads of GSE52248 for normal vs AIS, normal vs invasive lung tissues samples were passed to MuTect2 [25]. The normal samples were used as controls in the comparisons to obtain somatic mutations. We further collected the mutation profiles (VCF format) of 84 stage III lung adenocarcinoma cases from TCGA as comparable invasive tumor samples. After the PASS filtering of MuTect2, the resulting somatic mutations were fed into CHASM-5.2 [9], an online tool that calculates the mutation scores and then reports the putative driver genes. The lung adenocarcinoma was used as the disease type for prediction. The somatic mutations with score > 0.8 and P-value < 0.05 were predicted as putative driver mutations. The CHASM score ranged from 0 (likely passenger) to 1 (likely driver). The P-value is an empirical value representing probability that a passenger mutation is misclassified as a driver.

### Global subnetworks construction by GA

For each seed gene, the genes that interacted directly or indirectly with it through protein-DNA (TF and target genes) interactions and PPIs were considered as the candidate genes of the network. The maximum radius of the network from the seed gene was set as three. The unexpressed genes (median FPKM < 1) were removed from the candidate gene sets. The non-redundant PPIs were collected from five databases: intAct, MINT, BioGrid, DIP, and Reactome [26–30]. The lung cancer specific transcription factor and target gene pairs were downloaded from Regulatory Circuits [31]. GENIE3 [11] was applied to infer the target genes of the lncRNAs. GENIE3 adopts Random Forest to predict the regulatory relationships between genes according to the expression levels. The top 200 target genes that were potentially regulated by the lncRNAs were selected for network construction.

The R package genalg [32] was used for performing the GA analysis. We used the binary GA (0 represents the correspond gene is unselected, whereas 1 means the genes is selected) to search the optimum subnetwork member genes. The length of the chromosome is equal to the number of the candidate subnetwork genes for each seed. The mutation rate was set as 5%, and the argument zeroToOneRate of the genalg was 19 for controlling the gene selection. A larger zeroToOneRate value results in a smaller number of genes remained in each generation. To find the core subnetwork, the subnetworks that did not contain any of putative driver genes were removed first. Then, we calculated the frequency of each gene in the remaining subnetworks and filtered out the genes with low frequency. Here, the cutoff frequency was set as 50%. We further conducted two statistical tests to evaluate the significance of subnetworks compared to genomic background. We constructed two null distributions of fitness scores through permutation of the sample labels and randomly selected network members, respectively, for 1000 times. Then, we calculated the corresponding P-values for each core networks to assess its significance.

## Additional files

Additional file 1: **Figure S1**. The hierarchical clustering of the 18 normal (blue), AIS (orange), and invasive samples (purple) based on gene expression. (XLSX 50 kb)

Additional file 2: **Table S1.** Invasive specific genes. (PDF 12 kb)

Additional file 3: **Figure S2.** The hierarchical clustering of the TCGA LUAD samples. Normal (blue) and stage III (purple). (PDF 50 kb)

Additional file 4: **Table S2.** GO terms of the up- and down-regulated genes. (XLSX 41 kb)

Additional file 5: **Figure S3.** The distribution of the best fitness scores of the GA searching in 500 generations. Each line represents the scores of one of the seed genes. (PDF 17 kb)

Additional file 6: **Table S3.** The enriched pathways of the 19 core subnetworks. (XLSX 38 kb)

Additional file 7: **Figure S4.** The p-values of the 19 core subnetworks. (PDF 160 kb)

Additional file 8: **Figure S5.** The KEGG VEGS signaling pathway. (PDF 46 kb)

Additional file 9: **Figure S6.** The 19 core subnetworks. The node in yellow and square is a putative drive gene predicted by CHASM. The node in orange stands for a transcription factor. The node in green or red represents an either down-regulated or up-regulated invasive-specific gene. (PDF 676 kb)

## Abbreviations

AIS: Adenocarcinoma in situ
BAC: Bronchioloalveolar carcinoma
CHASM: Cancer-specific High-throughput Annotation of Somatic Mutations
DE: Differential expression
DEGs: Differentially expressed genes
EMT: Epithelial-mesenchymal transition
FPKM: Fragments Per Kilobase of transcript per Million mapped reads
GA: Genetic Algorithm
lncRNAs: Long non-coding RNAs
LUAD: Lung adenocarcinoma
NSCLC: Non-small-cell lung cancer
PPIs: Protein-protein interactions
RNA-seq: RNA sequencing
TCGA: The Cancer Genome Atlas

## References

[1] Morton ML, Bai X, Merry CR, Linden PA, Khalil AM, Leidner RS (2014). Identification of mRNAs and lincRNAs associated with lung cancer progression using next-generation RNA sequencing from laser micro-dissected archival FFPE tissue specimens. Lung Cancer Amst Neth.

[2] Travis WD, Brambilla E, Riely GJ (2013). New pathologic classification of lung Cancer: relevance for clinical practice and clinical trials. J Clin Oncol.

[3] Travis WD, Brambilla E, Noguchi M, Nicholson AG, Geisinger KR, Yatabe Y (2011). International association for the study of lung cancer/american thoracic society/european respiratory society international multidisciplinary classification of lung adenocarcinoma. J Thorac Oncol off Publ Int Assoc study. Lung Cancer.

[4] Detterbeck FC, Jantz MA, Wallace M, Vansteenkiste J, Silvestri GA (2007). Invasive mediastinal staging of lung Cancer. Chest.

[5] Franklin WA (2000). Diagnosis of lung Cancer. Chest.

[6] Min JH, Lee HY, Lee KS, Han J, Park K, Ahn M-J (2010). Stepwise evolution from a focal pure pulmonary ground-glass opacity nodule into an invasive lung adenocarcinoma: an observation for more than 10 years. Lung Cancer.

[7] Keshamouni VG, Michailidis G, Grasso CS, Anthwal S, Strahler JR, Walker A (2006). Differential protein expression profiling by iTRAQ−2DLC−MS/MS of lung Cancer cells undergoing epithelial-mesenchymal transition reveals a migratory/invasive phenotype. J Proteome Res.

[8] Huang DW, Sherman BT, Lempicki RA (2008). Systematic and integrative analysis of large gene lists using DAVID bioinformatics resources. Nat Protoc.

[9] Carter H, Chen S, Isik L, Tyekucheva S, Velculescu VE, Kinzler KW (2009). Cancer-specific high-throughput annotation of somatic mutations: computational prediction of driver missense mutations. Cancer Res.

[10] Barnes L, Weltgesundheitsorganisation, International Agency for Research on Cancer, editors. Pathology and genetics of head and neck tumours: ... Reflects the views of a working group that convened for an editorial and consensus conference in Lyon, France, July 16–19, 2003. Reprinted. Lyon: IARC Press; 2007.

[11] Huynh-Thu VA, Irrthum A, Wehenkel L, Geurts P (2010). Inferring regulatory networks from expression data using tree-based methods. PLoS One.

[12] Ma J, Sawai H, Ochi N, Matsuo Y, Xu D, Yasuda A (2009). PTEN regulate angiogenesis through PI3K/Akt/VEGF signaling pathway in human pancreatic cancer cells. Mol Cell Biochem.

[13] He D, Li L, Zhu G, Liang L, Guan Z, Chang L (2014). ASC-J9 suppresses renal cell carcinoma progression by targeting an androgen receptor-dependent HIF2 /VEGF signaling pathway. Cancer Res.

[14] Kanehisa M, Furumichi M, Tanabe M, Sato Y, Morishima K (2017). KEGG: new perspectives on genomes, pathways, diseases and drugs. Nucleic Acids Res.

[15] Walesky C, Apte U (2015). Role of hepatocyte nuclear factor 4α (HNF4α) in cell proliferation and Cancer. Gene Expr.

[16] Cooper GM (2000). The cell: a molecular approach. 2. ed.

[17] Chen P-H, Chen X, He X (2013). Platelet-derived growth factors and their receptors: structural and functional perspectives. Biochim Biophys Acta BBA - Proteins Proteomics.

[18] Wei T, Zhang L-N, Lv Y, Ma X-Y, Zhi L, Liu C, et al. Overexpression of platelet-derived growth factor receptor alpha promotes tumor progression and indicates poor prognosis in hepatocellular carcinoma. Oncotarget. 2014;5. 10.18632/oncotarget.2537. 10.18632/oncotarget.2537 PMC4279374 25333264

[19] Chellappa K, Robertson GR, Sladek FM (2012). HNF4α: a new biomarker in colon cancer?. Biomark Med.

[20] Bolger AM, Lohse M, Usadel B (2014). Trimmomatic: a flexible trimmer for Illumina sequence data. Bioinformatics.

[21] Kim D, Pertea G, Trapnell C, Pimentel H, Kelley R, Salzberg SL (2013). TopHat2: accurate alignment of transcriptomes in the presence of insertions, deletions and gene fusions. Genome Biol.

[22] Anders S, Pyl PT, Huber W (2015). HTSeq—a Python framework to work with high-throughput sequencing data. Bioinformatics.

[23] Trapnell C, Roberts A, Goff L, Pertea G, Kim D, Kelley DR (2012). Differential gene and transcript expression analysis of RNA-seq experiments with TopHat and cufflinks. Nat Protoc.

[24] Robinson MD, McCarthy DJ, Smyth GK (2010). edgeR: a Bioconductor package for differential expression analysis of digital gene expression data. Bioinformatics.

[25] Cibulskis K, Lawrence MS, Carter SL, Sivachenko A, Jaffe D, Sougnez C (2013). Sensitive detection of somatic point mutations in impure and heterogeneous cancer samples. Nat Biotechnol.

[26] Orchard S, Ammari M, Aranda B, Breuza L, Briganti L, Broackes-Carter F (2014). The MIntAct project--IntAct as a common curation platform for 11 molecular interaction databases. Nucleic Acids Res.

[27] Chatr-aryamontri A, Ceol A, Palazzi LM, Nardelli G, Schneider MV, Castagnoli L (2007). MINT: the molecular INTeraction database. Nucleic Acids Res.

[28] Stark C (2006). BioGRID: a general repository for interaction datasets. Nucleic Acids Res.

[29] Salwinski L (2004). The database of interacting proteins: 2004 update. Nucleic Acids Res.

[30] Croft D, Mundo AF, Haw R, Milacic M, Weiser J, Wu G (2014). The Reactome pathway knowledgebase. Nucleic Acids Res.

[31] Marbach D, Lamparter D, Quon G, Kellis M, Kutalik Z, Bergmann S (2016). Tissue-specific regulatory circuits reveal variable modular perturbations across complex diseases. Nat Methods.

[32] Willighagen E, Genalg MB (2005). R based genetic algroithm. R Package Version 01.

