# Supplementary figures and images for: Systems biology analysis reveals new insights into invasive lung cancer
Source: BMC Syst Biol. 2018 Dec 14;12(Suppl 7):117. doi: 10.1186/s12918-018-0637-z (PMC6293490; doi:10.1186/s12918-018-0637-z)

Expression of Normal vs. Stage III

Normal Stage III

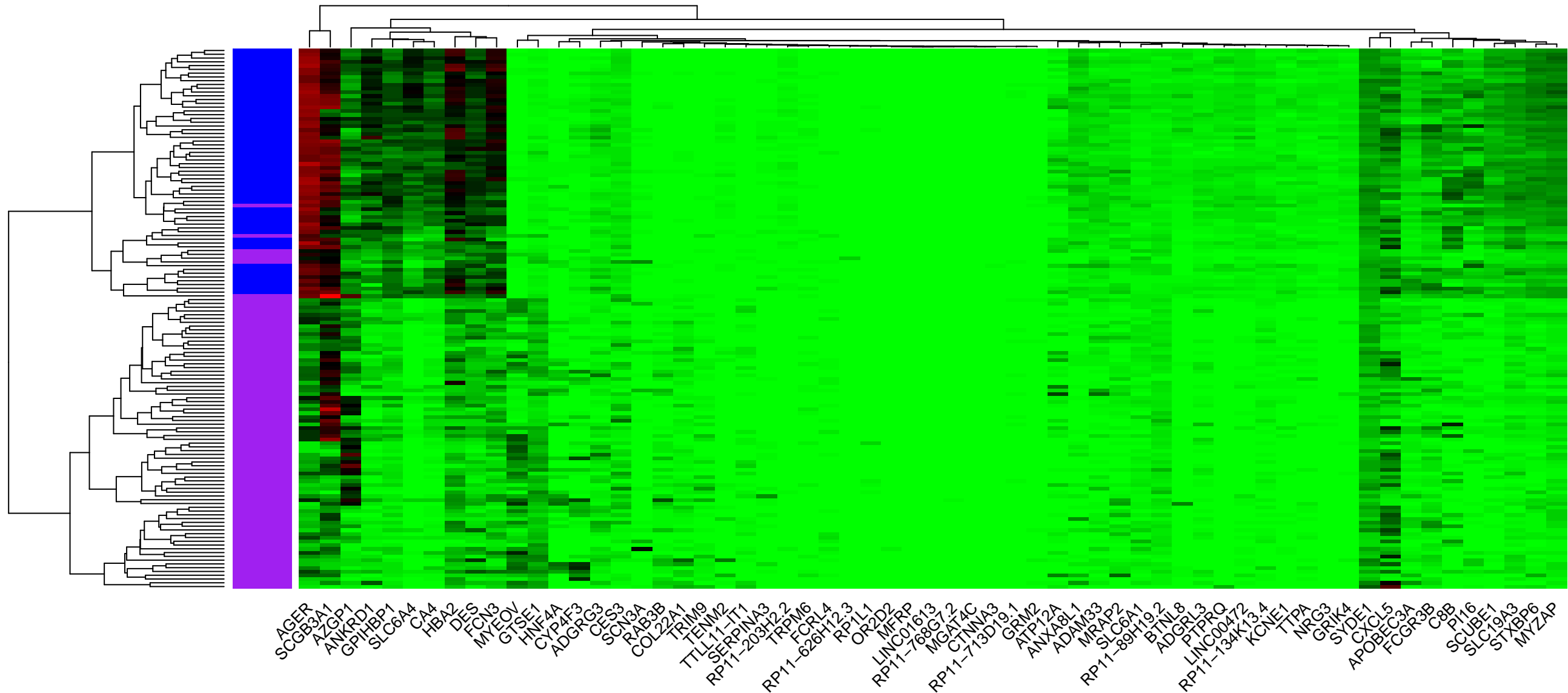

Supplement: Supplementary file 3 — Figure S2. The hierarchical clustering of the TCGA LUAD samples. Normal (blue) and stage III (purple). (PDF 50 kb) [file 12918_2018_637_MOESM3_ESM.pdf]

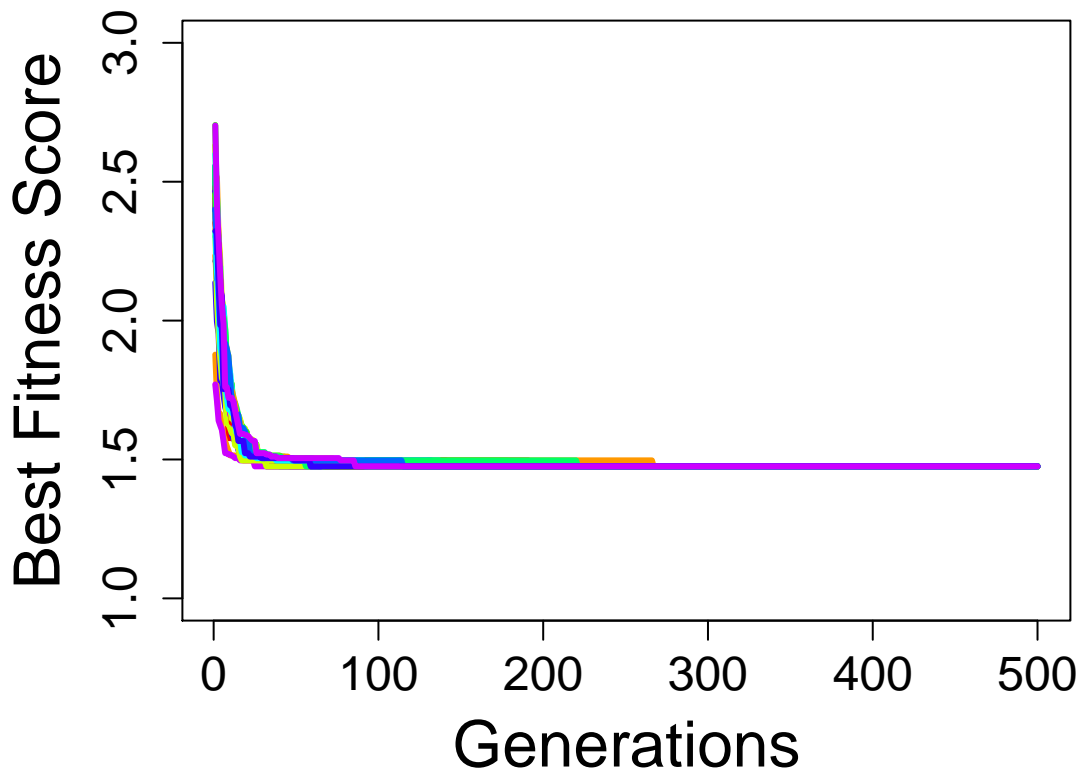

Supplement: Supplementary file 5 — Figure S3. The distribution of the best fitness scores of the GA searching in 500 generations. Each line represents the scores of one of the seed genes. (PDF 17 kb) [file 12918_2018_637_MOESM5_ESM.pdf]

## P-values of core subnetworks (1000 random tests)

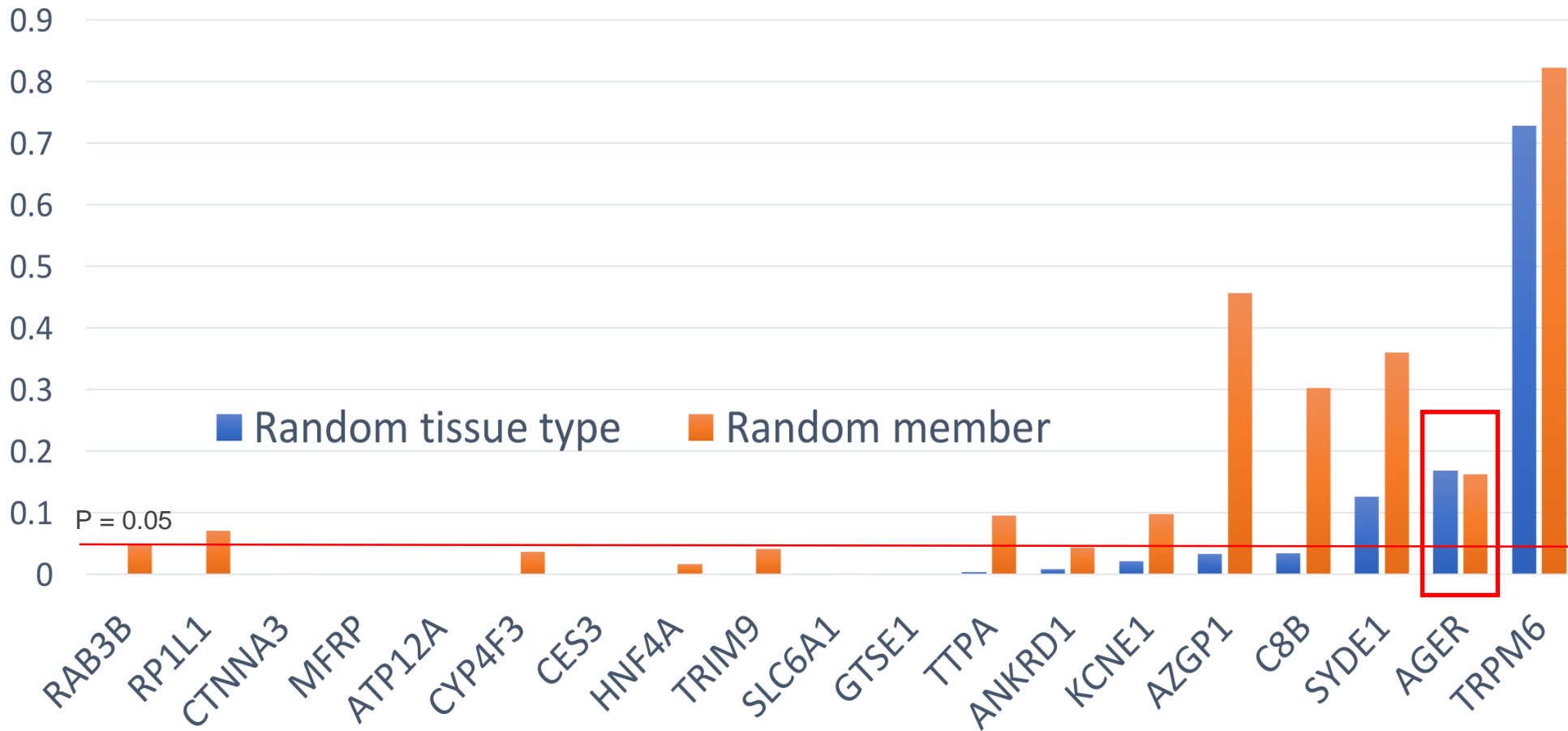

Supplement: Supplementary file 7 — Figure S4. The p-values of the 19 core subnetworks. (PDF 160 kb) [file 12918_2018_637_MOESM7_ESM.pdf]

# VEGF SIGNALING PATHWAY

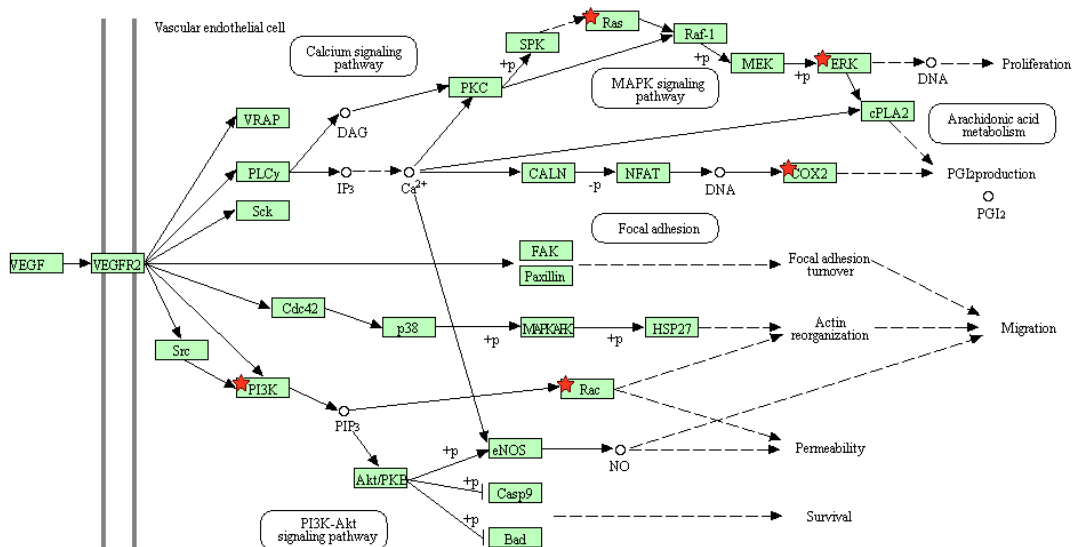

Supplement: Supplementary file 8 — Figure S5. The KEGG VEGS signaling pathway. (PDF 46 kb) [file 12918_2018_637_MOESM8_ESM.pdf]

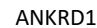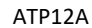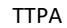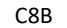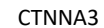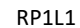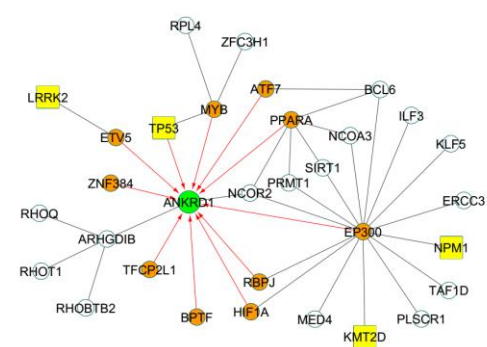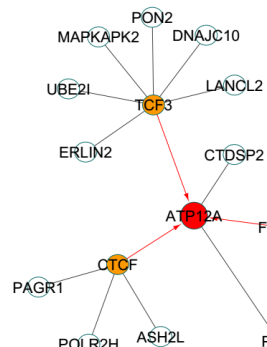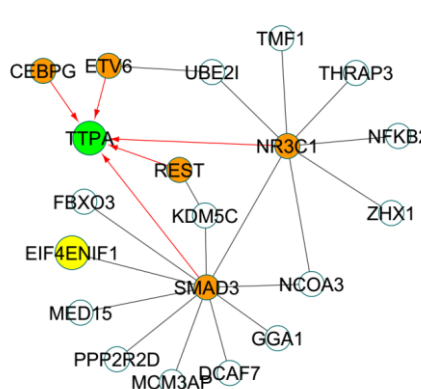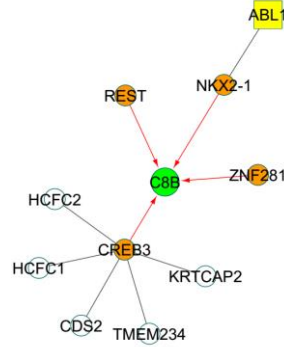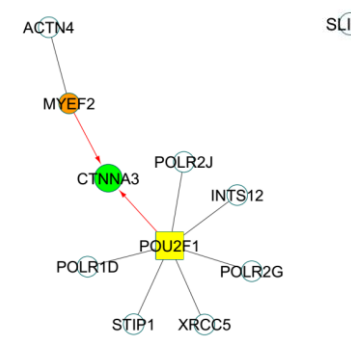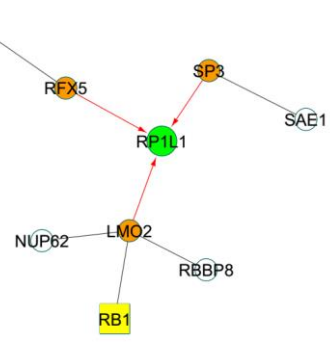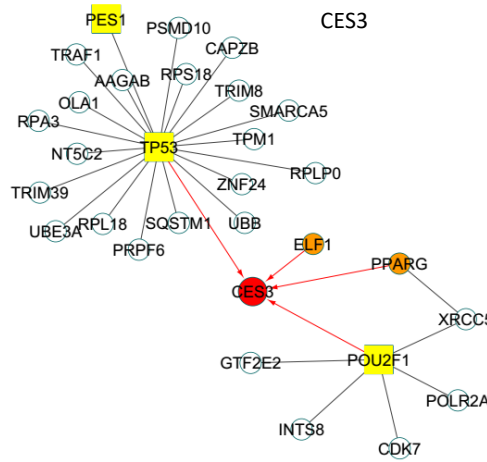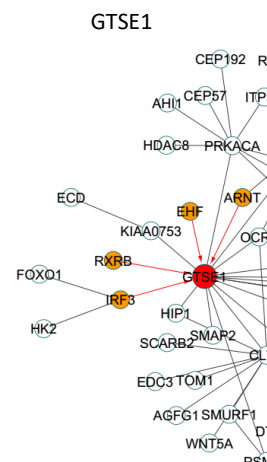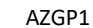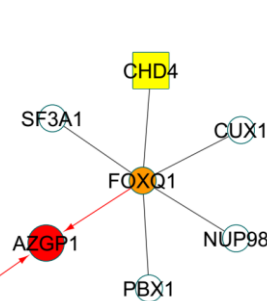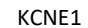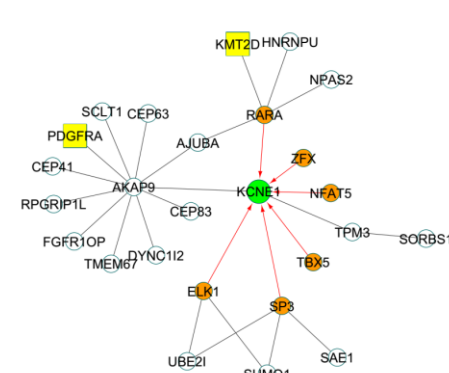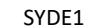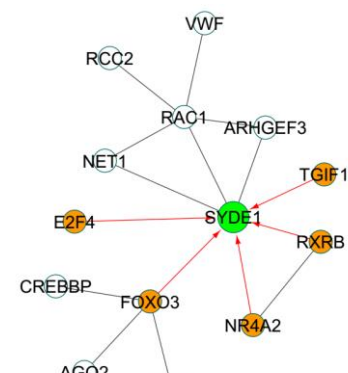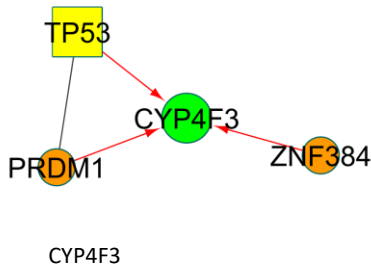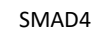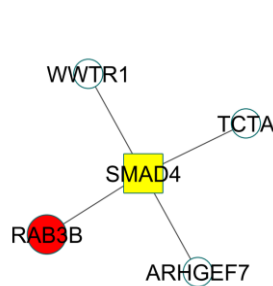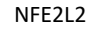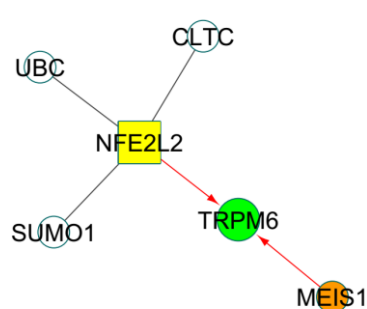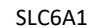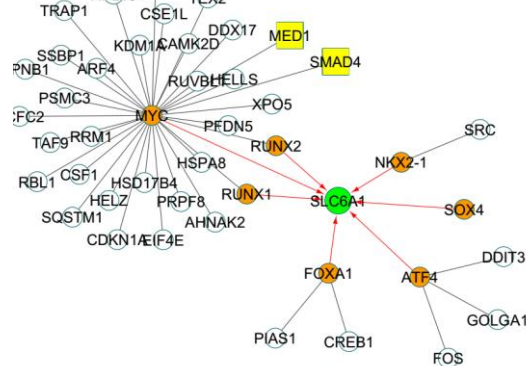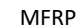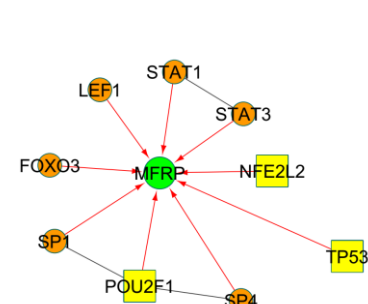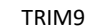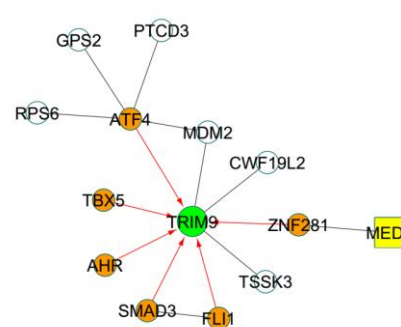

Supplement: Supplementary file 9 — Figure S6. The 19 core subnetworks. The node in yellow and square is a putative drive gene predicted by CHASM. The node in orange stands for a transcription factor. The node in green or red represents an either down-regulated or up-regulated invasive-specific gene. (PDF 676 kb) [file 12918_2018_637_MOESM9_ESM.pdf]
