# Supplementary material for: Systems biology analysis reveals new insights into invasive lung cancer
Source: BMC Syst Biol. 2018 Dec 14;12(Suppl 7):117. doi: 10.1186/s12918-018-0637-z (PMC6293490; doi:10.1186/s12918-018-0637-z)

Expression of AIS data

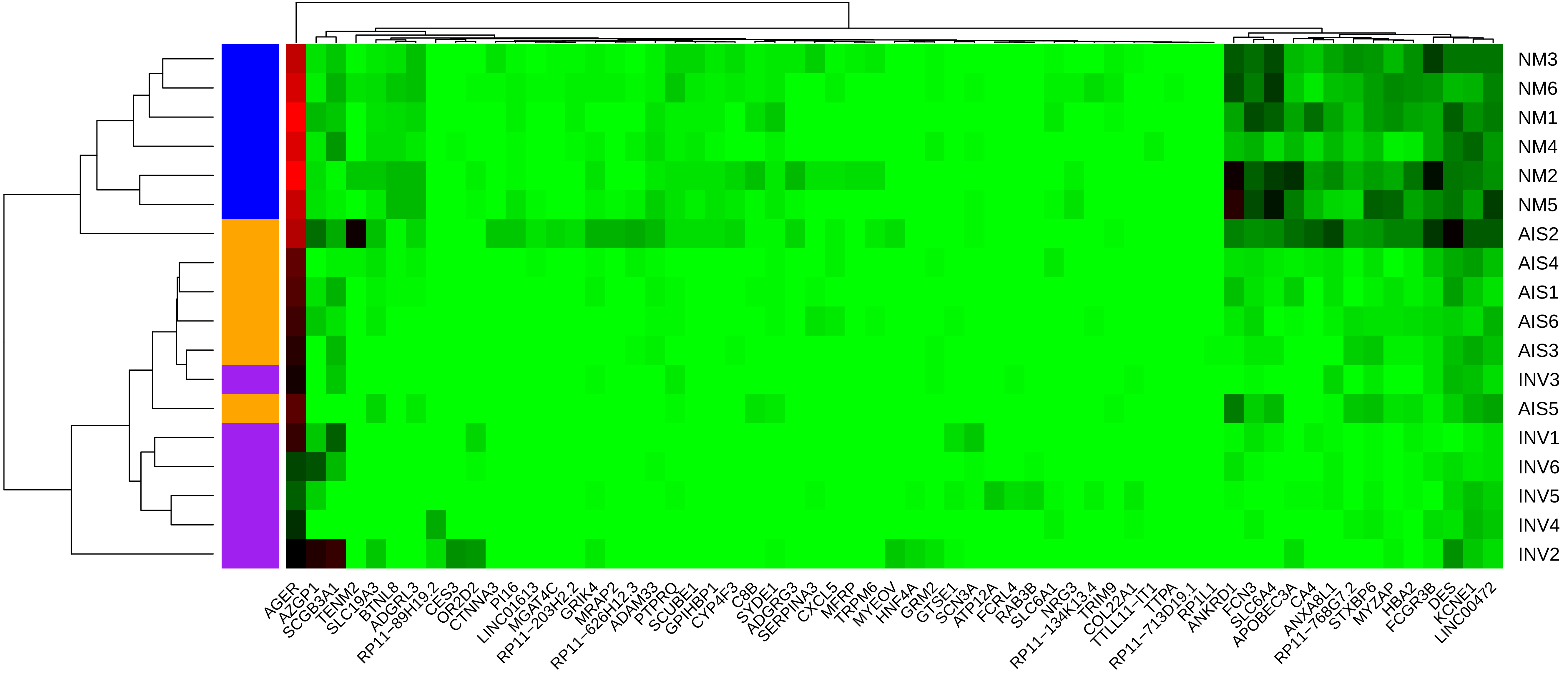

Supplement: Supplementary file 2 — Table S1. Invasive specific genes. (PDF 12 kb) [file 12918_2018_637_MOESM2_ESM.pdf]
